# Supplementary material for: Humoral SARS-CoV-2 vaccine responses are durable in solid organ transplant recipients with and without HIV
Source: medRxiv. 2025 May 8:2025.05.07.25327192. Preprint. [Version 1] doi: 10.1101/2025.05.07.25327192 (PMC12083590; doi:10.1101/2025.05.07.25327192)
Supplement: 1 — Supplemental Figure 1: Summary of the TITAN study participants data included in the different experiments This Sankey diagram illustrates the selection and classification of participants through various figures in the paper. The initial pool consists of 133 participants in the TITAN study. These participants were subsequently filtered into different figures based on various criteria. All active or completed participants are shown in Figure 1. Participants with transplant prior to vaccine dose 3 are shown in Figure 3, and of those participants with a pre-dose 3 and peak post-dose 3 timepoint are included in Figure 2. For Figure 5, 24 participants with 4–5 longitudinal follow-up timepoints were selected for additional experiments measuring microneutralization and RBD ACE2 binding inhibition (cPass). [file NIHPP2025.05.07.25327192V1-supplement-1.pdf]

medRxiv preprint doi: https://doi.org/10.1101/2025.05.07.25327192; this version posted May 8, 2025. The copyright holder has placed this preprint (which was not certified by peer review) in the Public Domain. It is no longer restricted by copyright. Anyone can legally share, reuse, remix, or adapt this material for any purpose without crediting the original authors.

Rana et al., Supplemental Figure 1

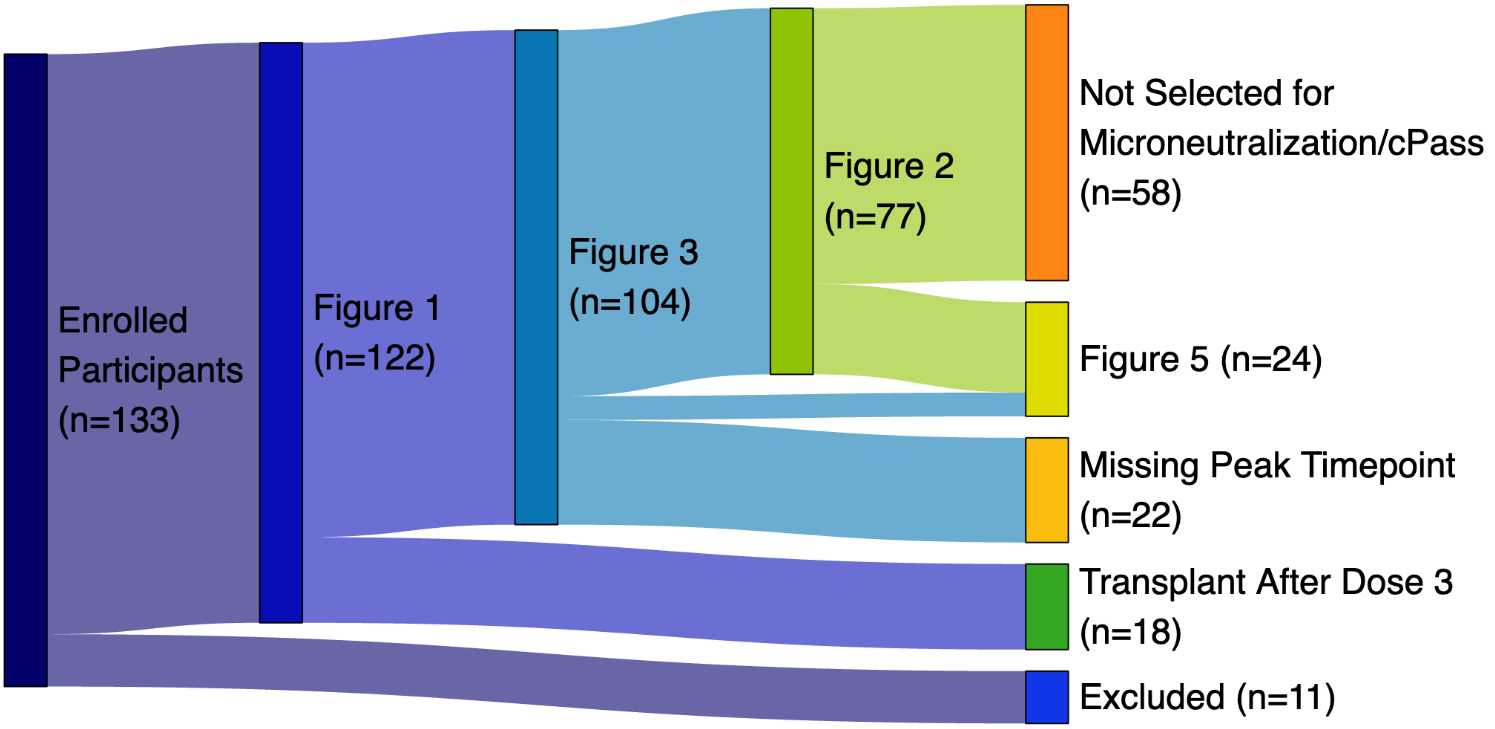

<u>Supplemental Figure 1</u>: Summary of the TITAN study participants data included in the different analyses. This Sankey diagram illustrates the selection and classification of participants from whom data were visualized in the five figures of this report. The initial group consists of 133 TITAN participants. Data from these participants were subsequently filtered into different figures based on various criteria.

All active or completed participants are shown in Figure 1.

Participants with transplant prior to vaccine dose 3 are shown in Figure 3, and of those participants with a pre-dose 3 and peak post-dose 3 timepoint are included in Figure 2.

For Figure 5, 24 participants with 4-5 longitudinal follow-up timepoints were selected for additional experiments measuring microneutralization and RBD ACE2 binding inhibition (cPass).
